# The practical impact of postgraduate leadership education on Registered Nurses’ practice: a narrative inquiry

**DOI:** 10.1177/17449871261465539

**Published:** 2026-08-06

**Authors:** Ashley Page, Jenny Sim, Elizabeth Halcomb, Christopher Patterson

**Affiliations:** PhD candidate, School of Nursing, University of Wollongong, Wollongong, NSW, Australia; Head of School and Professor, School of Nursing, Midwifery & Paramedicine (NSW), Australian Catholic University, North Sydney, NSW, Australia; Professor of Primary Health Care Nursing, School of Nursing, University of Wollongong, Wollongong, NSW, Australia; Associate Professor, School of Nursing, University of Wollongong, Wollongong, NSW, Australia

**Keywords:** clinical practice, education, leadership, narrative inquiry, nursing

## Abstract

**Background:**

Postgraduate leadership education is increasingly recognised as essential for preparing nurses to navigate the complexities of contemporary healthcare. However, there remains limited empirical understanding of how nurses integrate leadership learning into everyday clinical practice and the contextual factors that shape its enactment.

**Aims:**

This paper examines the practical impact of a postgraduate leadership course on Registered Nurses’ clinical practice.

**Methods:**

Clandinin and Connelly’s three-dimensional narrative inquiry space guided this study. Eleven Registered Nurses who had completed a postgraduate leadership course participated in two semi-structured interviews. Data were analysed using cross-narrative comparison to identify recurring threads and divergent experiences.

**Results:**

The practical impact of postgraduate leadership education was reflected in three interconnected narrative threads. ‘Identification of a skills gap’ described participants’ recognition of previously limiting leadership deficits. ‘Using the resources provided’ showed how leadership tools, frameworks and strategies were applied in practice. ‘Challenges of enacting education into practice’ highlighted contextual constraints that limited some participants’ ability to apply their learning despite strong motivation.

**Conclusion:**

Postgraduate leadership education can strengthen nurses’ clinical practice by enhancing leadership capability and readiness. However, organisational hierarchies, role clarity and structural support significantly influence the extent to which leadership learning can be enacted in practice.

## Introduction

Effective nursing leadership is central to the delivery of safe, high‑quality healthcare and is associated with improved patient outcomes, workforce stability, and team functioning (Alsadaan et al., 2023; Ergün Arslanlı et al., 2025). As healthcare environments become increasingly complex, nurses across all roles are expected to demonstrate leadership capability, extending beyond direct patient care to include quality improvement, governance and system‑level influence (Jiang, 2024). These expectations reflect a shift in how nursing leadership is conceptualised, from a function of formal management roles to a relational, practice‑based capability enacted within everyday clinical work (Kvist, 2026).

However, many of the leadership expectations placed on nurses in contemporary practice exceed the preparation typically provided within pre‑registration nursing education (Costa et al., 2025). In response, postgraduate leadership education has emerged internationally as a strategy to support nurses in developing leadership capability. Such programmes are designed to provide theoretical foundations, leadership frameworks and applied learning experiences intended to strengthen professional practice and organisational influence (Carson et al., 2023; Middleton et al., 2024). Evidence suggests that engagement in postgraduate leadership education is associated with improvements in communication, critical thinking, emotional intelligence and professional confidence, as well as increased perceived readiness to participate in decision‑making and improvement processes (Phillipson et al., 2025; Svetic Cisic et al., 2025).

Despite these reported benefits, there remains limited empirical understanding of how learning from postgraduate leadership education is enacted within nurses’ everyday clinical practice. Existing research has largely focused on perceived skill development or leadership competence, rather than examining how nurses translate postgraduate leadership education into practical action across diverse clinical and organisational settings (Page et al., 2021). In particular, there is insufficient insight into how nurses apply leadership knowledge in routine practice, how they recognise and address ongoing leadership development needs and how organisational structures, role boundaries and context influence the enactment of leadership learning (Aydogdu, 2023; Cummings et al., 2021; Fennell et al., 2025). Addressing this gap is important for understanding the practical value of postgraduate leadership education and its contribution to sustained leadership capacity in nursing practice.

## Method

### Aim

This paper explores the practical impact of postgraduate leadership education on Registered Nurses’ clinical practice. It reports findings from a broader Doctoral Study that examined how nurses embed leadership learning from a postgraduate leadership course into everyday clinical practice. Although the wider study focused on the processes through which leadership learning was integrated into practice, this paper specifically examines the practical enactment of leadership learning.

### Design

This study was informed by narrative inquiry (Clandinin and Connelly, 2000). Narrative inquiry explores human experience through the collection and interpretation of stories (Clandinin and Caine, 2012). Ontologically, narrative inquiry recognises experience as relational, contextual and temporal, shaped through continuous interactions between individuals, others and their environments (Caine et al., 2013). Epistemologically, it is grounded in the understanding that knowledge is constructed and conveyed through storytelling, with participants and researchers collaboratively interpreting experience to co-create meaning (Clandinin and Connelly, 2000).

### Recruitment and sampling

Advertisements were distributed via social media platforms (e.g. LinkedIn, Facebook) and professional organisations (e.g. Australian College of Nursing) to recruit participants. Nurses who expressed interest were invited to participate if they met the inclusion criteria. Participants were also encouraged to share study information with colleagues.

Eligible participants were baccalaureate‑prepared (or equivalent) Registered Nurses employed in any nursing role across Australia who had completed a postgraduate nursing leadership course within 3 years prior to recruitment. This timeframe was selected to ensure participants had sufficient opportunity to apply leadership learning in practice, while being able to provide recent and detailed reflections. Participant selection focused on recruiting nurses with direct and relevant experience of postgraduate leadership education to support in‑depth exploration of the study aim (Malterud et al., 2016).

### Data collection

Two semi-structured individual interviews were undertaken with each participant via Zoom. Video conferencing is widely accepted in qualitative research (Self, 2021) as it minimises travel requirements and accommodates participants’ schedules. Interviews were undertaken by the primary author (AP), a PhD candidate and female registered nurse with experience in leadership education.

The interview schedules were informed by narrative inquiry principles (Clandinin and Connelly, 2000) and guided by key concepts from the Knowledge‑to‑Action (KTA) Framework (Graham et al., 2006). The KTA Framework was used to shape interview prompts related to recognising leadership knowledge, adapting learning to clinical contexts and identifying barriers and enablers to implementation. The first interview began with an open, invitational question designed to elicit participants’ reflections on postgraduate leadership education and its application in clinical practice, including leadership development, its enactment in everyday work and the influences on learning transfer. The second interview built upon initial discussions, prompting deeper reflection on the outcomes of learning and its practical application.

All interviews were audio-recorded and transcribed verbatim. Interviews lasted between 19–99 minutes each (mean 59 minutes). A participant number was used to de-identify transcripts, while still allowing the two transcripts for each participant to be linked. Field notes were taken during and immediately after each interview to capture contextual details, non-verbal cues and the researcher’s reflections. Data collection continued until sufficient information power was achieved, such that the depth and relevance of participant accounts were adequate to address the study aim (Malterud et al., 2016).

### Data analysis

Interview transcripts were read and re‑read in full and initially coded to ensure familiarity with each participant’s account. Analysis was guided by Clandinin and Connelly’s (2000) three‑dimensional narrative inquiry space, with data examined in relation to temporality (past, present and future experiences), sociality (personal and interpersonal contexts) and place (clinical and organisational settings). This analytic lens provided a structured way of attending to how participants experienced and interpreted learning from postgraduate leadership education over time and within specific practice contexts.

Using a template to guide analysis, transcripts were then examined to identify key moments, significant events and recurring patterns within and across stories. These narrative elements informed the development of interpretive categories and emergent narrative threads, forming a preliminary coding framework grounded in participants’ accounts. NVivo software (Lumivero, 2025) was used to support data management, including organising transcripts, coding narrative segments and collating illustrative quotations.

Coding and interpretation were primarily conducted by the primary author (AP), with ongoing reflexive discussion of analytic decisions and emerging interpretations with the research team. These discussions supported consistency of interpretation and enhanced the credibility of the analysis.

### Ethical considerations

Informed consent was gained from each participant prior to data collection. Participants were able to withdraw from the study at any time without consequence, however, none did.

### Rigour

Rigour was maintained using the criteria of credibility, dependability, confirmability and transferability (Lincoln and Guba, 1985). Credibility was enhanced through prolonged engagement with participants across two interviews, iterative reading of transcripts and regular supervisory debriefing to refine interpretations. Dependability was supported by maintaining an audit trail, documenting methodological decisions, analytic processes and reflexive notes. As the primary author (AP) has a vested interest in leadership education, their positionality was acknowledged throughout the study, with careful attention to how prior experiences and assumptions may have shaped data generation and interpretation. Confirmability was strengthened through ongoing reflexivity, in which the authors critically examined these assumptions, their positionality and potential influence on the research process. Transferability was facilitated by providing rich, contextualised descriptions, enabling readers to determine applicability to other contexts.

## Results

### Participant characteristics

The 11 participants were aged from 28 to 56 years and had 6 to 35 years nursing experience. Reflecting the gender disparity in the workforce, only 2 (18.1%) participants were male. Participants were employed in diverse roles, including direct patient care (*n* = 3), nurse education (*n* = 2) and management (*n* = 6) roles with responsibilities for service delivery, coordination, quality improvement and strategic leadership. Participants were geographically distributed across five of the eight Australian State/Territories. Participants had completed postgraduate leadership qualifications, including Graduate Certificates, Graduate Diplomas and Masters’ programmes, offered by various education providers. Programmes were primarily delivered online (*n* = 9), with some blended formats (*n* = 2).

### Threads

Three interrelated threads capture how leadership knowledge and skills developed through postgraduate education were taken up, applied and sometimes constrained in everyday clinical practice. These threads were, ‘Identification of a skills gap’, ‘Using the resources provided’ and ‘Challenges of enacting education into practice’. Together, these threads highlight both the potential and the complexity of embedding leadership learning into clinical work, illustrating the varied and sometimes uneven practical impacts experienced by participants.

#### Identification of a skills gap

Prior to undertaking postgraduate study, reliance on instinct or habit was frequently described, alongside a limited understanding of leadership roles and the broader implications of actions. Alex’s reflections offer one example of how the course prompted a retrospective awareness of long-standing leadership gaps: ‘I realised I probably didn’t believe in my capabilities as much as I thought I did. . . the gap for me was the leadership knowledge—the actual research leadership knowledge. And I didn’t actually realise that pre-course’.

For Alex, this recognition extended across clinical decision-making, communication, systems thinking and organisational leadership. Alex described previously ‘getting through the day’ on an operational level without stepping back to assess quality, risk or performance: ‘I used to turn up to work, do the bed meeting, find the beds, roll around and go home’.

Postgraduate leadership education provided learning that directly addressed the participants’ skills or knowledge gaps. For Cameron, this structured learning was transformative: ‘With this course, I’m more self-assured that the decisions I’m making are correct. . . I’m more confident in my decision-making that what I’m doing is right’. Other participants echoed this sense of practical transformation. Francis highlighted improvements in her ability to write persuasively, advocate effectively and influence decision-makers:

It changes the way you write a briefing note or escalate for equipment or resources. It changes the way you advocate and argue to improve practice. . . because your ability to write or articulate is better, because you have this leadership journey. (Francis)

Similarly, Bailey described how the education gave her the tools to gather and analyse data to drive local improvement: ‘Those units around ward clinical governance and accountability. . . really supported me to understand where to find the resources and collect the data I needed to demonstrate that I could improve our current practice’.

Content on topics such as quality and safety, communication, financial management, clinical governance and leadership theory were described as both relevant and immediately applicable to practice. Alex described how the course expanded her professional capabilities, introducing areas that had previously felt inaccessible: ‘We run $1.5/2 million budgets, and we did a finance subject in the postgrad. That was all new to me, but now I approach budgets with a very different perspective’. She went on to describe how they ‘influence a lot of policy now’ and is asked ‘to participate in new policy development. . . and I write those’.

For some participants, the education provided a long-term foundation for professional growth. As Cameron reflected: ‘What I have learnt is completely invaluable, and these are skills that I will be able to take no matter what area of nursing I work in’. Francis similarly described changes in her professional practice, particularly in written communication with decision‑makers: ‘I’ve seen a very big difference in the way I write since doing the Master’s. . . and how to present information in a way that gets a one- or two-pass approval rather than backwards and forwards’.

#### Using the resources provided

The second thread illustrates how participants drew on a range of leadership resources, including leadership styles, coaching-oriented approaches and quality improvement methods, to guide everyday clinical practice. In some instances, the timing of leadership learning aligned closely with organisational priorities, enabling theory to be translated into action. As Cameron reflected: ‘It was quite convenient at times, particularly when we were doing human resource management. When we were actually beginning to strategic plan in the workforce’.

It was described how the course gave them the confidence and capability to take on new organisational roles or projects. Emerson explained that the course directly prompted her to join a cytotoxic management working group: ‘I don’t think I would’ve joined that team without. . . doing my leadership course’. This ability to connect course content with organisational needs strengthened confidence in implementing change.

Ongoing access to course materials further supported the application of learning, and participants regularly revisited content to guide decisions and problem‑solving in practice. Cameron kept printed manuals at work as a reference point, whereas Bailey noted frequent use of course learning to support daily decision‑making:

I actually have my manuals printed at work. [Laughs] So I’ve got some home copies and I actually have the copy at work as well. They sit in my drawer at work so if there’s ever something I need to think about I can go back to that. (Cameron)

I probably, almost daily, I refer back to stuff that I have learnt in the course to support decisions that I’m making. (Bailey)

Similarly, Alex described building a personal ‘toolkit’ of conceptual and practical resources that she could draw upon: ‘In my toolkit, I have communication skills, emotional intelligence. . . quality, safety, my team. . . whatever challenge comes my way, I feel like I can face it’.

Learning about leadership styles supported more intentional leadership practice. Kendall described how the education enabled her to actively select and adapt her approach to different situations: ‘Learning about different leadership styles. . . and then actively choosing which style I wanted to be’. Indy reinforced this view, explaining that postgraduate education expanded awareness of multiple leadership styles and enabled deliberate selection of leadership approaches ‘depending on the situation’, rather than adherence to a single, fixed leadership style. Georgie similarly described how engagement with leadership theory provided through her course reinforced the situational nature of leadership, noting that postgraduate education highlighted that ‘there’s not necessarily one leadership style. . . you use multiple throughout the course of every day’. Together, these accounts illustrate how learning about leadership styles and applying this learning into their context enabled participants to support conscious, adaptive leadership practice rather than reliance on a single, fixed approach.

Coaching‑oriented approaches supported more constructive responses to staff development and performance challenges. Drew explicitly described applying coaching strategies when addressing performance concerns, noting a clear shift away from previous management practices: ‘I was able to effectively coach some people at work. . . we had performance issues, and I was actually able to coach them rather than the old way I would’ve done it’. For Indy, this shift was reflected in a move away from directive management towards more facilitative and negotiated leadership practices. Rather than instructing staff, he described working collaboratively to address challenges: ‘. . .not just telling people, “This is what you’re going to do,” but actually negotiating with them’.

Quality improvement principles were also widely used. While conducting a learning needs analysis, Cameron identified critical gaps in staff knowledge and used these insights to develop targeted education: ‘We did a learning needs analysis and that actually helped us develop a. . . training program on washer racks. . . it’s also been able to identify the knowledge gaps in what we’re teaching staff as well’. Similarly, Bailey described applying a structured, evidence‑informed approach to clinical problems, moving beyond ad‑hoc solutions towards research‑guided practice change:

Rather than just going, ‘That’s another cream’ and put that on and see what happens, it’s following that academic type approach. . . looking at the research. . . interpreting the research. . . and recognising how to get the rest of the team along to actually achieve what it is that we’re trying to achieve. (Bailey)

Drew described applying course learning to a practice‑based project that aligned with workplace priorities. The discharge‑planning project was developed in response to local needs and was well received within the clinical setting: ‘The project we did, we did something that was relevant to work and that was received really well. . . We did ours on preparing residents to go home’.

#### Challenges of enacting education into practice

The final thread examines the implementation gap between acquiring leadership knowledge and enacting it in clinical practice. Although leadership education equipped participants with valuable tools, frameworks and ways of thinking, enactment was frequently constrained by role boundaries, limited authority and organisational context. Participants whose current roles lacked formal managerial responsibility described delays or restrictions in applying what they had learned.

Harrie articulated this constraint succinctly, explaining that leadership education: ‘helped me learn all these new things, but if I don’t work at that level, I’m not really using that’. Participants described their current role as a major factor influencing their capacity to apply course content:

In my current role. . . I couldn’t apply it at all. . . The course itself isn’t designed to be at a clinical level. (Harrie)

If I was a manager I definitely would’ve been able to put that into practice. (Emerson)

When you go and try and put them into action, if you’re not directly in that kind of role, then it’s very difficult to apply some of those things because you don’t have permission. (Indy)

Bailey’s account further illustrates how limited professional authority and recognition constrained the enactment of leadership learning. Reflecting on her early role, she described feeling powerless and lacking credibility: ‘I got very burnt out in that role because I think I was probably a bit too junior. . . I really struggled with the [Medical Specialist] and getting them to try and take me seriously’. This lack of perceived authority made it difficult to apply leadership learning related to clinical governance and influence within the team. Her experience reflects a broader pattern within the data, whereby participants possessed leadership knowledge and intent but encountered structural and relational barriers that constrained enactment in practice.

Despite these challenges, participants did not view leadership learning as redundant or static. Several described postgraduate education as a longer‑term investment that shaped how they interpreted situations, even when immediate enactment was not possible. Indy noted that learning could be drawn upon in less direct ways, explaining: ‘It may not be right there in your face, but you can always find other ways of being able to apply that knowledge’. Jordan similarly described enactment as a gradual, context‑dependent process, emphasising the role of experience and exposure: ‘You’d have to be exposed to the scenarios. . . and then you’re like, “OK, yes, I can relate back to that”’. This process mirrors reflective practice, in which leadership knowledge is consolidated and enacted incrementally as opportunities emerge.

## Discussion

The findings of this study provide insight into how postgraduate leadership learning is translated into clinical practice, addressing the study’s aim of understanding the impact of leadership education in real‑world settings. In doing so, they extend existing knowledge by illustrating how participants move beyond knowledge acquisition to enact and adapt learning within their clinical contexts. These processes demonstrate clear conceptual alignment with key elements of the KTA framework, particularly the application and integration of knowledge into practice, while recognising that the framework serves here as a point of reference rather than an analytic guide.

Despite its strengths, this study had some limitations. Firstly, participants were drawn from those who responded to a call for participants. As such, they may differ from the broader cohort of nurses who have completed postgraduate nursing leadership education. Secondly, participants had completed diverse programmes and had varying work experience. While this provides diversity, it must be considered when interpreting study findings. As the study was qualitative and not designed to evaluate the structure or content of postgraduate programmes, links cannot be made between specific pedagogical approaches, participants’ experiences and outcomes. Finally, as a qualitative study, research data were drawn from self-reports and relied on participants’ recall of past behaviours and actions. However, the diversity of participants and the richness of their stories are key strengths of the study.

Participants in this study described how they translated postgraduate leadership learning into a practical, reusable ‘toolkit’ that supported their everyday clinical decision-making. The capacity to revisit course materials, apply frameworks reflectively and intentionally select leadership approaches in response to situational demands indicates that leadership learning was not experienced as abstract or detached from clinical work, but as materially embedded within routine nursing practice. This finding reinforces existing evidence that structured leadership education enhances nurses’ confidence, self-efficacy and ability to engage intentionally with organisational systems, particularly where theory is explicitly connected to real-world clinical contexts and role-based responsibilities (Curtis et al., 2011; Middleton et al., 2021). For nurses working in complex healthcare environments, tangible leadership resources such as reusable frameworks appear to function as enduring cognitive scaffolds, supporting clinical decision‑making, reflective practice and problem‑solving beyond the formal education context (Masava et al., 2023). Participants’ active and ongoing use of leadership frameworks and quality improvement methodologies also signals a shift from reactive or ad‑hoc responses to clinical problems towards more systematic, evidence‑informed practice change. This transition aligns with contemporary clinical governance expectations, which position nurses as active contributors to quality, safety and continuous improvement through the use of structured leadership and evidence‑based practice frameworks (Melnyk and Fineout-Overholt, 2022). These findings are consistent with research showing that leadership education enhances the ability to implement system-level improvements, support team functioning and influence organisational outcomes (Välimäki et al., 2024). Overall, the emergence of a personalised leadership ‘toolkit’ aligns with contemporary conceptualisations of nursing leadership as adaptive, situational and relational, rather than hierarchical or trait based. By equipping nurses with both a conceptual vocabulary and practical resources, postgraduate leadership education fosters more intentional, confident and system-aware practice. These findings underscore the importance of leadership curricula that prioritise applicability, integration within workplace contexts and sustained access to learning resources.

This study also found that postgraduate leadership education enabled participants to identify gaps in their leadership knowledge and skills they had not previously recognised. Prior to undertaking formal study, leadership was commonly enacted in an instinctive, task‑focused or operational manner, with limited explicit engagement with governance, organisational risk, strategic decision‑making or influence across systems. Participants’ reflections suggest that while leadership responsibilities were already embedded in their roles, they were undertaken without a clear conceptual or theoretical foundation, aligning with evidence that nurses frequently assume leadership functions without adequate preparation or role clarity (Metersky and Fisher, 2025). In this study, postgraduate education prompted retrospective insight into these gaps, allowing participants to identify limitations in their leadership knowledge and understanding that had not been apparent beforehand. This finding aligns with qualitative research indicating that nurses often only recognise the extent of leadership knowledge deficits once they are exposed to structured leadership education that provides the language, frameworks and systems perspective necessary for critical interpretation of practice (Middleton et al., 2024). Importantly, these gaps were not experienced as a lack of motivation or capability, but as an educational deficit related to how leadership, governance and system accountability are conceptualised and taught (Institute of Medicine, 2011). Without such education, nurses may continue to function effectively at a task level while remaining underprepared for the broader leadership and improvement responsibilities increasingly expected in contemporary healthcare (Catrambone and Mohr, 2026; Marcellus et al., 2018).

This study highlights enduring tensions between the acquisition of leadership knowledge and the capacity to enact it in practice. For some participants, the translation of leadership learning was constrained by role boundaries, organisational structures and limited formal authority, resulting in delayed or partial enactment of leadership capabilities. This finding is consistent with evidence suggesting that leadership capability alone is insufficient without structural empowerment, clear role delineation and organisational support that legitimises leadership practice (Cummings et al., 2018; Wong et al., 2013). Research in nursing leadership consistently shows that even highly capable clinicians may struggle to exercise leadership influence in the absence of enabling conditions such as supportive governance structures, access to decision‑making forums and recognition of informal leadership roles (Kadosh and Rozani, 2025; Lenssen et al., 2025). Participants described gradually integrating their leadership learning as they encountered relevant clinical situations or transitioned into roles affording greater influence. This reflects an understanding of leadership development as iterative and cumulative, shaped by experience, mentorship and situated practice rather than discrete educational interventions (Geerts, 2024). Comparable findings in the literature suggest that leadership education reframes how clinicians interpret practice contexts and prepares them for future enactment, even where immediate application is constrained (Fosah and Llahana, 2025). Collectively, these findings indicate that postgraduate leadership education contributes to longer-term leadership capacity by enhancing clinicians’ readiness to lead, while also underscoring the critical importance of organisational pathways that enable leadership knowledge to be meaningfully activated in practice.

## Conclusion

This paper demonstrates that postgraduate leadership education plays a pivotal role in shaping how nurses understand, interpret and enact leadership within clinical practice. Through postgraduate education, leadership knowledge became embedded in routine practice, enabling more intentional, systematic and contextually informed responses to clinical and organisational challenges. Education also fostered critical awareness of previously unrecognised gaps in leadership knowledge and preparation, highlighting that, prior to formal study, leadership had often been enacted intuitively, without the conceptual language or frameworks required for systems-level influence. Leadership development emerged as an iterative, cumulative process, with education shaping readiness to lead even when immediate enactment was constrained.

**Key points for policy, practice and research**

- This study demonstrates that leadership education can impact nurses’ clinical practice.
- Strategies for implementing leadership knowledge and skills should be incorporated within leadership education programmes.
- Education providers should ensure that postgraduate leadership courses incorporate practical learning activities and specific guidance for nurses without leadership or management experience.
- Embedding structured opportunities for applied learning, reflection and ongoing support may help bridge the gap between acquiring leadership knowledge and translating it into practice settings.
- Designing learning experiences that emphasise transferable skills and real-world application can better prepare nurses to navigate diverse environments and organisational demands.
